# The relationship between autophagy and PD-L1 and their role in antitumor therapy

**DOI:** 10.3389/fimmu.2023.1093558

**Published:** 2023-03-15

**Authors:** Yu Cui, Jinfeng Shi, Youbin Cui, Zhanpeng Zhu, Wei Zhu

**Affiliations:** ^1^ Department of Otolaryngology, Head & Neck Surgery, First Hospital of Jilin University, Changchun, China; ^2^ Department of Thoracic Surgery, First Hospital of Jilin University, Changchun, China; ^3^ Department of Neurosurgery, First Hospital of Jilin University, Changchun, China

**Keywords:** PD-1, PD-L1, antitumor therapy, tumor escape, autophagy

## Abstract

Immune checkpoint blockade therapy is an important advance in cancer treatment, and the representative drugs (PD-1/PD-L1 antibodies) have greatly improved clinical outcomes in various human cancers. However, since many patients still experience primary resistance, they do not respond to anti-PD1/PD-L1 therapy, and some responders also develop acquired resistance after an initial response. Therefore, combined therapy with anti-PD-1/PD-L1 immunotherapy may result in better efficacy than monotherapy. In tumorigenesis and tumor development processes, the mutual regulation of autophagy and tumor immune escape is an intrinsic factor of malignant tumor progression. Understanding the correlation between the tumor autophagy pathway and tumor immune escape may help identify new clinical cancer treatment strategies. Since both autophagy and immune escape of tumor cells occur in a relatively complex microenvironmental network, autophagy affects the immune-mediated killing of tumor cells and immune escape. Therefore, comprehensive treatment targeting autophagy and immune escape to achieve “immune normalization” may be an important direction for future research and development. The PD-1/PD-L1 pathway is essential in tumor immunotherapy. High expression of PD-L1 in different tumors is closely related to poor survival rates, prognoses, and treatment effects. Therefore, exploring the mechanism of PD-L1 expression is crucial to improve the efficacy of tumor immunotherapy. Here, we summarize the mechanism and mutual relationship between autophagy and PD-L1 in antitumor therapy, which may help enhance current antitumor immunotherapy approaches.

## 1 Introduction

### 1.1 Autophagy and cancer cell immune escape

**Figure 1:**
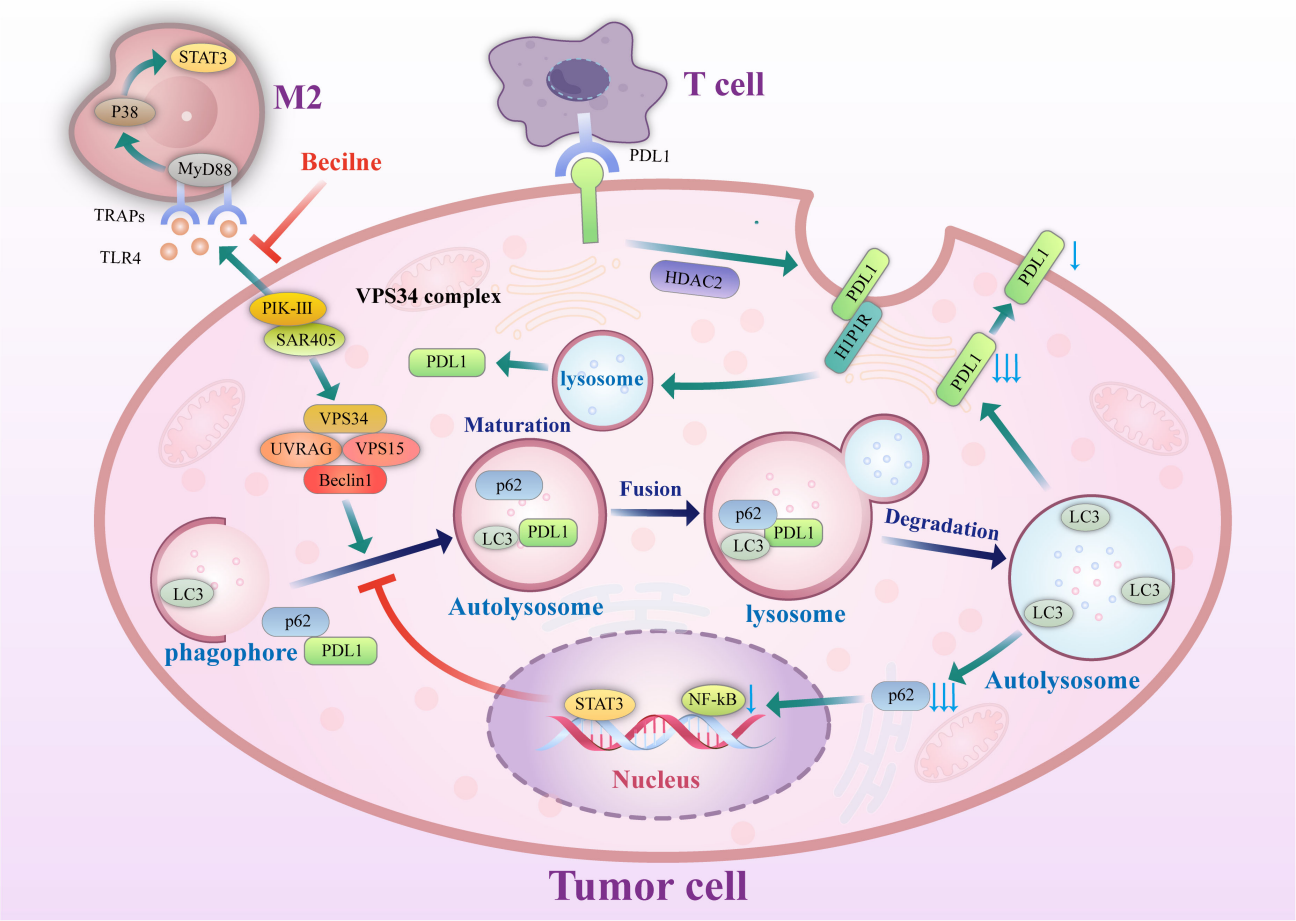
Autophagy and cancer cell immune escape.

Autophagy has been reported to be essential in regulating cancer cell immune escape (1), involving many aspects such as autophagy and PD-1/PD-L1, autophagy and MHC-I/MHC-II, mitophagy and tumor immune escape, autophagy and exosome, and so on (**Figure 1**). Valecka et al. reviewed the autophagy and MHC-restricted antigen presentation (2). MHC-I/II plays a vital role in antigen-presenting cells (APCs), while cancer cells can escape immune surveillance by degrading MHC-1 (3). MHC-I/II undergoes autophagic degradation in cancer cells by NBR1 or March 1, leading to tumor immune escape. In addition, AAK1 induces MHC-1 autophagic degradation in DCs, inhibiting antigen presentation and T-cell activation. Thus, autophagy regulates MHC-I/II stability, and autophagy inhibitors treatment enhances the efficacy of anti-tumor therapy (4). Lisanti et al. reviewed autophagy/mitophagy in the tumor microenvironment (5). Mitophagy is a selective autophagy process by the clearance of damaged or dysfunctional mitochondria (6). Mitophagy is essential in regulating the immune response against cancer (7). Mitophagy induction in STAT3-deleted cancer cells increases antigen presentation for DCs and T cell activation. In addition, PINK1/PARK2 or FUNDC1-mediated mitophagy promotes clearance of damaged mitochondria leading to increased antitumor immune response. Buratta et al. reviewed exosome release and secretory autophagy (8). Exosomes are cellular secreted vesicles which play an essential role in regulating crosstalk between cells (9). The immune checkpoint protein PD-L1 and CD47 are presented on exosomes, which maybe escape autophagic degradation in the lysosome (10). In this review, we aim to focus on only one aspect, the autophagy and PD-1/PD-L1, one of the most critical aspects in the immune escape.

Autophagy has been reported to play an essential role in regulating cancer cell immune escape, involving many aspects such as autophagy and PD-1/PD-L1, autophagy and MHC-I/MHC-II, mitophagy and tumor immune escape, autophagy and exosome, and so on.

### 1.2 Modulated autophagy for improved efficacy of immunotherapeutic treatment

Multiple potential strategies have been shown to modulate autophagy for improved efficacy of immunotherapeutic treatment (11) (**Table 1**). Radiotherapy and chemotherapy might provoke autophagy, contributing to enhanced effectiveness of immunotherapeutic treatment. Radiation or chemotherapy-induced autophagy can redistribute mannose-6-phosphate receptor (MPR) with its ligands to the autophagosomes (22). Autophagy functions in antigen processing for MHCI and MHCII presentation. Moreover, SYK augments OxLDL-induced autophagy and MHCII expression in macrophages. The OxLDL-induced and SYK-mediated autophagy facilitates the surface expression of MHCII and CD4+ T cell activation. SYK may enhance anti-tumor immunotherapy effects *via* autophagy-mediated adaptive immune responses (23). DCs-based vaccines exhibit promising therapeutic effects in promoting tumor immunotherapy. Shikonin-induced autophagy can directly contribute to damage-associated molecular patterns (DAMPs) upregulation and DCs activation (24). In addition, autophagy improves the efficacy of DNA vaccines by synthesizing intracellular vaccine-encoded tumor antigens (25). The regulatory pathways help target the autophagy pathway in tumor cells, making autophagy a promising target in cancer treatment. There are plenty of targeted autophagic proteins and autophagy inhibitors for cancer treatment. For example, autophagic proteins, including UVRAG (in initiation), BECN1 (in initiation), ATG5 (in elongation), and ATG2B (in fusion), play tumor suppressor roles. On the opposite, PDPK1 (in upstream), ATG4B (in elongation), ATG16L1 (in extension), and ULK1 (in initiation) play an oncogenic role.

**Table 1:** Clinical strategy of targeted autophagy in tumor therapy.

Tumour type	Autophagy inhibitor	Clinical trial phase	Combination
Brain metastases	CQ	II	Radiation (12)
glioblastoma multiforme	CQ	III	Radiation therapy and temozolomide (13)
glioblastoma multiforme	HCQ	I/II	Radiation therapy and temozolomide (14)
non-small cell lung cancer	HCQ	I/II	Carboplatin and Paclitaxel (15)
Pancreatic cancer	HCQ	II	Gemcitabine, nab-paclitaxel (16)
Solid tumours, including melanoma	HCQ	I	Temozolomide (17)
Solid tumours	HCQ	I	Vorinostat (18)
metastatic astration-resistant prostate cancer	Pantoprazole	II	Docetaxel (19)
Solid tumours	Pantoprazole	I	Doxorubicin (20)
Refractory myeloma	Ricolinostat	I/II	Bortezomib and Dexamethasone (21)

Autophagy is activated for protection by mediating the acquired resistance in some cancer cells during chemotherapy. Therefore, autophagy inhibitors could enhance the therapeutic effect of chemotherapy. Generally, there are four groups of autophagy-inhibiting molecules: (a) repressors of autophagosome formation, (b) repressors of lysosomal acidification, (c) inhibitors of autophagosome-lysosome fusion, (d) silencing the expression of autophagy-related proteins at the transcription level. Class III PI3K inhibitors 3-methyladenine (3-MA), Wortmannin, and SAR405 target PI3KC3, which blocks autophagosome formation. Chloroquine and Hydroxychloroquine target lysosomal pH, inhibiting cargo degradation in the autophagosomes. Spautin-1 targets the Beclin-1 subunit of Vps34 complexes, which inhibits ubiquitin-specific peptidases (26, 27).

### 1.3 PD-1/PD-L1 immune checkpoint pathway in tumor immunotherapy

The PD-1/PD-L1 immune checkpoint pathway is essential in tumor immunotherapy. Programmed death-1 (PD-1) is an immunoregulative costimulatory molecule mainly expressed on the surface of activated T cells, B cells, macrophages, and bone marrow cells. In contrast, programmed death-1 ligand (PD-L1) is described on the surface of tumor cells (28). Programmed death-ligand 1, which binds to PD-1, can inhibit T lymphocyte activation and induce T cell apoptosis by inhibiting the PI3K/AKT/mTOR. Ras/MEK/Erk signaling pathways (29), resulting in T cell “exhaustion” (30, 31). At the same time, the upregulation of PD-L1 on dendritic cells inhibits the secretion of IL-2, IL-10, INF-γ, INF-α and other cytokines by T lymphocytes in the tumor microenvironment, downregulates the immune “surveillance” function of T cells, and mediates tumor immune escape (32–34). PD-1 and PD-L1 can inhibit tumor-specific T cells (Ts, suppressor T cells) and promote the differentiation of regulatory T cells (Tregs). More importantly, the PD-1/PD-L1 pathway can activate the highly active PI3K/AKT/mTOR pathway of tumor cells and promote high intracellular glycolytic metabolism, which results in tumor cell survival (34, 35). Therefore, PD-1/PD-L1 immune checkpoint blockade can enhance the efficacy of tumor immunotherapy (36, 37). PD-1/PD-L1 inhibitors are used to block the PD-1/PD-L1 immune checkpoint pathway. On the one hand, these inhibitors can reawaken the “depleted” T-cell immune function and activate the activity of CD8+ cytotoxic T lymphocytes (CD8+ CTLs). This process “restores” the body’s immune system and inhibits the glycolytic metabolism of tumor cells to eliminate tumor cells (38, 39). On the other hand, PD-L1 activity on the tumor cell surface is reduced, and activation of the intracellular PI3K/AKT/mTOR pathway and inhibition of autophagy are also reduced.

In recent years, immune checkpoint inhibitors represented by PD-1/PD-L1 monoclonal antibodies have made breakthrough progress in tumor immunotherapy. In 2014, the FDA approved the first PD-1 inhibitors, pembrolizumab, and nivolumab, to treat refractory melanoma. Since then, various PD-1/PD-L1 inhibitors have been approved for the treatment of advanced head and neck malignancies (40), melanoma (41), Hodgkin’s lymphoma (42), non-small cell lung cancer (43), and other tumors, all of which have shown significant clinical benefits. However, in some tumor types, the clinical efficacy of drugs targeting PD1/PD-1 alone is poor (44), and even fulminant tumor progression occurs. Many patients experience primary drug resistance and do not respond to PD1/PD-L1 treatment. Some responders also develop acquired drug resistance after the initial reaction (45). Due to insufficient antigen immunogenicity, dysregulation of antigen presentation, irreversible T-cell exhaustion, resistance to IFN-γ signaling, and an immunosuppressive tumor microenvironment (TME), tumors can prevent the antitumor efficacy of T cells by forming an unfavorable TME, which leads to primary drug resistance and avoids tumor rejection. After an initial response to PD1/PD-L1 blockade therapy, some patients eventually develop resistance or relapse, and sometimes, the host immune system plays an important role in these events. Through cancer immunoediting, tumor cells that can escape antitumor immunity gradually become dominant. In addition, activation of the PD1/PD-L1-independent inhibitory pathway and redepletion of activated T cells can again disable T-cell function in the presence of PD1/PD-L1 blockade; that is, secondary drug resistance occurs. Therefore, the development of targeted solutions to improve the efficacy of anti-PD1/PD-L1 therapy is urgently needed. Factors that affect the response to PD-1/PD-L1 inhibition include the early localization of tumor infiltrating lymphocytes (TILs) (46) and activation level (47), as well as the influence of tumor cell mutations (48). In addition, the expression level of PD-L1 on the tumor cell membrane is positively correlated with the effect of targeted drugs, which is an important factor that determines therapeutic effect and prognosis. The high expression of PD-L1 in different tumors is closely related to poor survival rates, prognoses, and treatment effects (49, 50). In addition, the lack of or abnormal expression of PD-L1 will also lead to the ineffectiveness of PD-1/PD-L1 inhibitors (51). Therefore, PD-L1 can be used as a potential predictive marker for the efficacy of PD-1/PD-L1 inhibitors. The PD-L1 pathway can be used as an entry point to understanding the regulatory mechanism of PD-L1 to provide ideas for improving anti-PD-1/PD-L1 treatment.

It is generally believed that autophagy plays a tumor-suppressive role in the early stage of tumorigenesis and is conducive to the survival of tumor cells after tumor formation. Numerous studies have shown that autophagy activation in tumor cells is essential in weakening antitumor immune responses and that targeting autophagy can inhibit tumor growth. Therefore, inhibition of autophagy in tumor cells is a promising new method for cancer immunity. However, developing targeted drugs to inhibit autophagy is challenging because most autophagy-related proteins have multiple other effects besides autophagy. Understanding the mechanism of autophagy-related proteins and their impact on tumor immune function can provide a theoretical basis for the combination of autophagy-related protein inhibitors and immune checkpoint inhibitors.

## 2 Effects of autophagy regulation on PD-L1

PD-L1 is an immunosuppressive molecule expressed on the surface of tumor cells and on the membrane of various immune cells. PD-L1 plays an immunosuppressive role mainly by explicitly binding to PD-1 on the surface of T lymphocytes, which inhibits the proliferation of and induces the apoptosis of T lymphocytes (52, 53).

Autophagy is a self-digesting cellular process that separates cells from the cytoplasm by forming double-membrane vesicles (autophagosomes) that degrade cellular contents, including organelles and proteins, in a short period; this process thus enables cells to survive harsh conditions such as hypoxia and starvation. Autophagy is currently divided into three forms: 1) macroautophagy, 2) microautophagy, and 3) chaperone-mediated autophagy (CMA). Among them, macrophages are the dominant cell type that participates in autophagy, a standard process of degrading cytoplasmic components and organelles in the cell cycle to recover nutrients. Autophagy can reduce the expression of PD-L1 *in vivo* and *in vitro* (54). Autophagy has been demonstrated to play a dual role in regulating tumor immunity and treatment. Autophagy can positively or negatively regulate tumor immunotherapy by degrading immune checkpoints, regulating cytokine release, and modifying autoantigens on tumor cells. On the one hand, in the early stage of tumor development, as a mechanism of cell activity regulation, autophagy can remove damaged proteins, DNA, and organelles to maintain standard cell structure and function, thereby stimulating antitumor immune effects (55).

**Figure 2:**
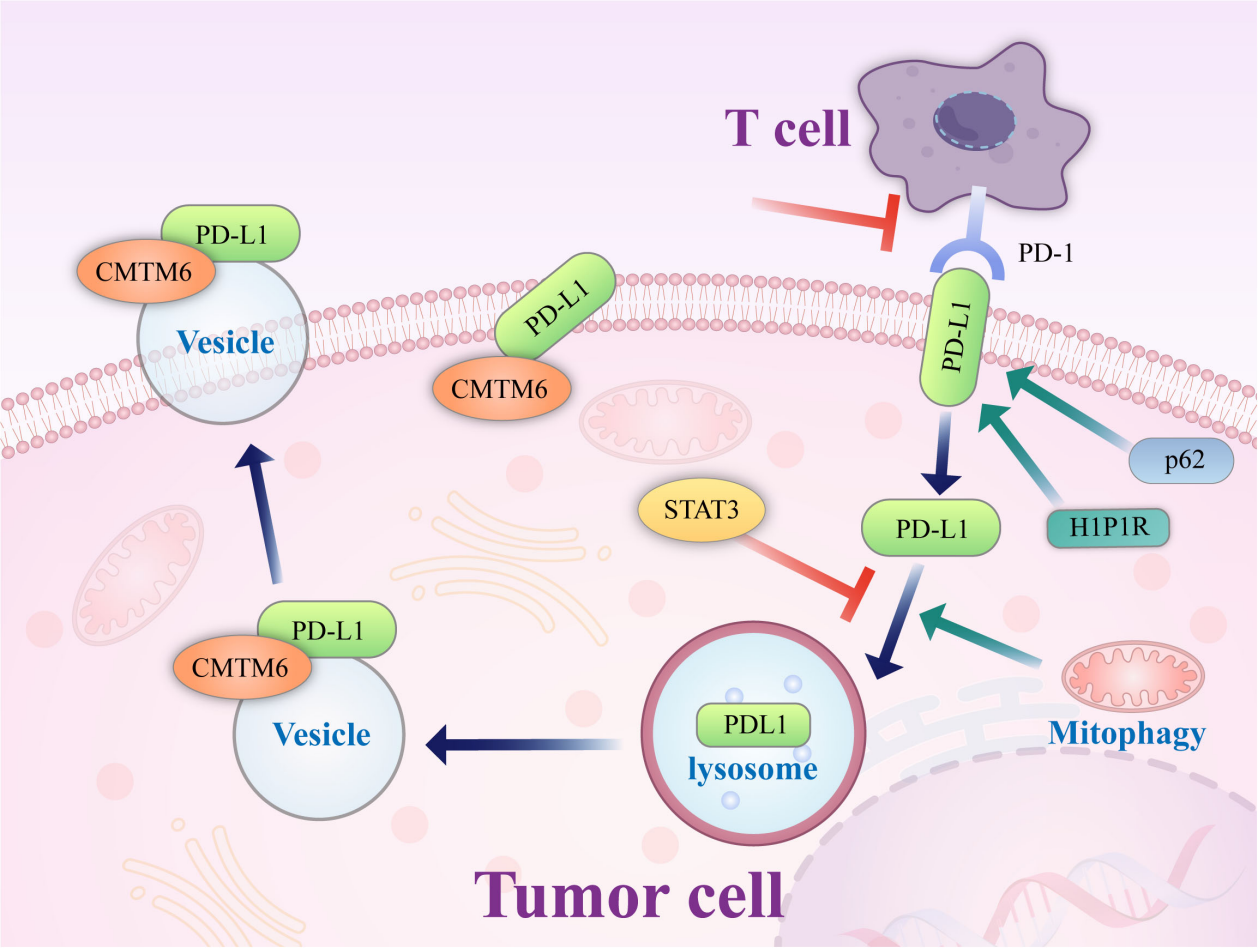
Mechanism of autophagy control. Autophagy controls PD-L1 expression *via* the histone deacetylases, p62/sequestosome-1/NF-κB pathway, STAT3 phosphorylation, ATG7/autophagy/FOXO3A/miR-145 axis, and autophagy flux in cancer cells. PD-1, programmed death-1; miRNA/miR, microRNA; PD-L1, programmed death ligand-1; p, Phosphorylated; HDAC, histone deacetylase.

On the other hand, autophagy can also play a negative role in the induction of tumor cell monitoring. Genetic inactivation of autophagy in tumor cells can also enhance the efficacy of immune checkpoint inhibitors in mouse tumor models (56), in which autophagy is activated in tumor cells by inhibiting mTOR signaling; this promotes tumor cell escape from T cell-mediated killing. The mechanism by which autophagy influences PD-L1 is shown in **Figure 2**.

### 2.1 Autophagy regulates the expression of PD-L1 through P62/SQSTM1

P62/SQSTM1 (abbreviated P62) is an essential multifunctional protein that regulates apoptosis, inflammatory response, cell survival, signal transduction, and tumor progression. P62 is a ubiquitin-binding protein closely related to protein ubiquitination and is involved in regulating a variety of cell signal transduction and autophagy processes. In autophagy, P62 binds to ubiquitinated proteins and then forms a complex with LC3-II protein localized on the autophagosome membrane; the ubiquitinated proteins and P62 are degraded together in lysosomes. Therefore, when autophagy occurs, the P62 protein is continuously degraded in the cytoplasm; when autophagy activity is weakened, or autophagy function is defective, the P62 protein accumulates in the cytoplasm. P62 is a marker protein that reflects autophagy activity, and its level indirectly reflects the clearance level of autophagosomes. P62-mediated selective autophagy and the subsequent activation of NF-κB and other tumor-stimulating signaling pathways may be one of the initiating factors of tumor formation. Studies have reported (57) that autophagy in gastric cancer regulates the expression of PD-L1 through the P62/SQSTM1-/NF-κB signaling pathway. In 137 primary gastric cancer specimens, LC3 and P62/SQSTM1 protein levels were positively correlated with the expression of PD-L1. Autophagy inhibition by autophagy inhibitors and small interfering RNA increases the expression level of PD-L1 both *in vitro* and *in vivo*, which may be related to the accumulation of P62 and increased NF-κB activation caused by inhibition of autophagy. After autophagy is inhibited, P62 can still maintain tumor development by promoting the activation of downstream pathways such as NF-κB, and thus, P62 and autophagy cooperate to support tumor growth. In addition, the expression levels of P62 and autophagy are closely related to the response to chemotherapy. Multiple studies (58, 59) have shown that P62 levels in platinum-resistant ovarian epithelial cancer cells are significantly increased, which is related to the activation of the NF-κB pathway by P62, and a reduction of P62 levels by an autophagy inducer can dramatically increase the sensitivity of ovarian epithelial cancer to platinum. Therefore, Leidal et al. (60) believed that autophagy levels and P62 levels should be inhibited in treating some tumors.

### 2.2 Targeting Vps34 enhances the effect of immune checkpoint inhibitors of PD-1 and PD-L1

Vacuolar protein Sorting 34 (Vps34) is a type III. Vacuolar Protein Sorting 34 (Vps34) is a type III phosphoinositide 3-kinase (PIK3C3). Vps34 is a critical protein in the process of autophagy. Vsp34 combines many autophagy-related proteins, including Vps15, Beclin 1, and Atg14, and by a complex interaction, Vps15/Atg14/UVRAG/Beclin1 controls the formation of autophagosomes and their translocation. Vsp34 is essential for the initiation of autophagy (61, 62) and can therefore serve as a potential target to inhibit autophagy (63, 64) from improving the tumor immune efficacy of PD-1/PD-L1 immune checkpoint disruption (65). Studies have found (66)that decreased numbers of immune cells and their dysfunction in the tumor microenvironment are essential mechanisms of the immune escape of tumor cells. Knockdown of Vps34 protein levels or inhibition of Vps34 activity induces the expression of the chemokines CCL5 and CXCL10 through the STAT1-IRF7 axis, which increases the capacity of tumor tissues to recruit immune cells. Through the effect of a Vps34 inhibitor, the tumor’s immunogenicity and immune cell infiltration in the tumor microenvironment can be enhanced. Moreover, combining antibodies to the immune checkpoints PD-1 and PD-L1 with Vps34 inhibitors yielded better therapeutic effects in a mouse tumorigenesis model. This is a novel idea that may further improve the efficacy of immunotherapy.

### 2.3 Inhibition of autophagy disrupts TRAP formation and leads to a significant delay in tumor growth

Tumor cell-released autophagosomes (TRAPs) is used as an immunosuppressive mechanism by tumor cells (67). Macrophages are transformed into an immunosuppressive M2-like phenotype characterized by increased expression of PD-L1 and IL-10 through the TLR4-mediated MyD88-P38-STAT3 signaling pathway, which inhibits the proliferation of CD4+ and CD8+ T cells *in vitro* and promotes tumor growth mainly through PD-L1 *in vivo*. *In vivo* studies have shown that disruption of TRAP formation by silencing the autophagy gene Beclin1 leads to a significant delay in tumor growth, associated with decreased autophagosome secretion, tumor-associated macrophage (TAM) reprogramming, and enhanced T-cell activation. This result provides a solid theoretical basis for targeting autophagy as a therapeutic approach to improve the efficacy of anti-PD-1 or anti-PD-L1 tumor immunotherapy.

### 2.4 Inhibition of autophagy upregulates PD-L1 expression by promoting STAT3 phosphorylation

STAT3 was first discovered as an oncogene regulating cell growth, differentiation, apoptosis, and other physiological pathways. Phosphorylated STAT3 binds to the promoters of target genes and activates transcription. Under the stimulation of oncogenic signals, STAT3 is continuously activated, is constitutively expressed in the nucleus in an activated state, continuously activates target genes, and promotes tumor cell growth (68, 69). Tammy et al. demonstrated that STAT3 activation could upregulate PD-L1 expression, which may be a potential mechanism that supports the immune escape of tumor cells. A close relationship between the STAT3 pathway and tumor autophagy has been established. Activation of the STAT3 pathway or STAT3 overexpression can inhibit autophagy, while STAT3 dephosphorylation can significantly increase the level of autophagy (70).Similarly, inhibition of autophagy can increase STAT3 activity. Tang et al. (71)found that STAT3 was phosphorylated by miRNA-3127-5P, which inhibited autophagosome formation and led to the upregulation of PD-L1 expression in NSCLC cells; therefore, this miRNA plays a vital role in immune escape and chemotherapy resistance in lung cancer.

### 2.5 The degradation of PD-L1 is promoted by increasing autophagic flux

Studies have found (65) that PD-L1 exists not only on the surface of tumor cells but also on the intracellular Golgi apparatus and external vesicles. PD-L1 on cancer cells can inhibit tumor immune escape, promote tumorigenesis, and supplement inactivated PD-L1 on the cell surface. This may be one of the reasons for the failure of PD-L1 antibody-based drugs (72). Autophagic flux is a dynamic and continuous concept that covers the entire process of autophagosome formation, transport of autophagic substrates to lysosomes, and degradation of autophagosomes within lysosomes. Autophagic flux is an index that reflects autophagic activity. As an autophagy receptor bound to PD-L1, Huntingtin interacting protein one associated protein (HIP1R) exerts a significant negative regulatory effect on PD-L1, which can promote the autophagic degradation of PD-L1 by lysosomes, reduce the level of PD-L1 and increase the immune killing effect of T cells. In cell-based starvation assays, HIP1R loss decreased autophagic flux (73) and increased PD-L1 protein levels (72). The integrated membrane scaffold protein SIGMA I stabilizes and enhances PD-L1 in tumor cells by preventing its autophagic degradation to a large extent (74), and in triple-negative breast cancer and androgen-independent prostate cancer cells expressing SIGMA I, the interaction between SIGMA I and glycosylated PD-L1 results in the inhibition of PD-L1 autophagic degradation. PD-L1 protein levels were inhibited by RNAi knockdown of SIGMA I and slight molecule inhibition of SIGMA I, as demonstrated by the use of the SIGMA inhibitor [1-(4-chlorophenyl)-3 -(2-adamantyl) guanidine] (IPAG) alone.

IPAG can induce autophagy waves, increase LC3B expression, decrease PD-L1 expression on the cell surface, and increase T-cell activity, which indicates that autophagy can promote PD-L1 degradation (74).

### 2.6 Autophagic degradation of histone deacetylase downregulates PD-L1 expression

Studies have confirmed (75) that PD-L1 is transported from the plasma membrane to the nucleus by interacting with components of endocytic and nucleoplasmic transport pathways, a process regulated by P300-mediated PD-L1 acetylation and histone deacetylase 2 (HDAC2)-dependent deacetylation. Inhibition of PD-L1 acetylation by genetic or pharmacological approaches prevents its nuclear translocation and promotes the reprogramming of immune genes, enhancing the antitumor response to PD-1 blockade. Pharmacological and genetic deletion of HDAC2 enhances PD-L1 acetylation. Degradation of HDACs by pemetrexed + sildenafil is dependent on autophagy. In one study, lung and ovarian tumor cells were exposed to pemetrexed + sildenafil *in vitro*, and knockdown of essential autophagy-related proteins (AMPKα, Beclin1, or ATG5) blocked the expression of various HDACs (HDAC2, HDAC4, HDAC6, and HDAC9). In contrast, the HDAC inhibitors AR42 and valproate enhanced the killing ability of pemetrexed + sildenafil. This suggests that exposure of tumor cells to pemetrexed + sildenafil would result in tumor cell death, autophagy-dependent downregulation of HDAC and PD-L1, and opsonization of the remaining tumor cells into targeted antitumor immunotherapy antibodies (76).

## 3 Effect of PD-L1 on autophagy

In addition to immune pathogenesis, the PD1/PD-L1 signaling pathway also plays a crucial role in tumors’ intrinsic function and survival (77). Innate PD-L1 signaling in tumor cells regulates mTOR, autophagy, growth, metastasis, drug resistance (e.g., to small molecules, chemotherapy, immunotherapy), epithelial to mesenchymal transition, and DNA damage repair in various types of tumor cells (77–81). Recent experimental results of mouse melanoma cells and human ovarian cancer cells showed that compared with cells with weak PD-L1 receptor expression, cells with high PD-L1 receptor expression were more sensitive to autophagy inhibitors (82). In one study of human ovarian cancer tissues, PD-L1 was overexpressed and promoted autophagy of ovarian cancer cells by upregulating the expression of BECN1 (83), a key molecule in autophagy regulation. Other studies have also shown that autophagy inhibitors have no significant negative impact on the immune system (84). This finding provides a potential opportunity to use autophagy inhibitors in PD-L1-overexpressing cells as a new avenue for cancer treatment. This study provides an experimental basis for the further exploration of the PD-L1 signaling pathway and the autophagy mechanism of different cell types. It helps to determine whether autophagy inhibitors combined with anti-PD-L1 treatment can enhance the clinical antitumor response.

Tumors adapt to resource deprivation through different survival mechanisms, of which autophagy is one of the most important. Although autophagy is beneficial to normal cells, under cancer conditions, it helps malignant cells adjust and adapt to unfavorable environments so that they develop and continue to grow. In addition, autophagy also plays an immunomodulatory role. Blockade of the PD-L1/PD1 axis by antibodies, such as those against PD1 or PD-L1, triggers autophagy in tumor cells, which allows nearby cells to recycle nutrients and signals the release of cytokines and extracellular vesicles. Therefore, with the widespread use of immune checkpoint inhibitors, autophagy regulation of the immune system has become the research focus. Selective blockade of the PD-1/PD-L1 immune checkpoint based on the autophagy signaling pathway can effectively improve the efficacy of tumor immunotherapy. However, although the autophagy inhibitors and PD-L1-resistant agents used in combination provide opportunities for enhanced antitumor activity, autophagy inhibition affects immune system-mediated tumor cell death as part of a complex channel network, and further experiments are needed to guide this synergy, especially in the initial stage of tumor progression.

## Author contributions

WZ and ZZ designed the study. YC wrote the manuscript. JS and YC consulted relevant materials and drew pictures. All authors contributed to the article and approved the submitted version.
